# Safety and Activity of Metronomic Temozolomide in Second-Line Treatment of Advanced Neuroendocrine Neoplasms

**DOI:** 10.3390/jcm8081224

**Published:** 2019-08-15

**Authors:** Salvatore Tafuto, Claudia von Arx, Monica Capozzi, Fabiana Tatangelo, Manuela Mura, Roberta Modica, Maria Luisa Barretta, Antonella Di Sarno, Maria Lina Tornesello, Annamaria Colao, Alessandro Ottaiano

**Affiliations:** 1 Department of Abdominal Oncology, Istituto Nazionale Tumori, IRCCS-Fondazione “G. Pascale”, 80131 Naples, Italy; 2 ENETs (European NeuroEndocrine Tumors Society), Center of Excellence of Naples, 80131 Naples, Italy; 3 Department of Surgery and Cancer, Imperial College London, London W12 0HS, UK; 4 Department of Pathology, Istituto Nazionale Tumori, IRCCS-Fondazione “G. Pascale”, 80131 Naples, Italy; 5 Department of Clinical Medicine and Surgery, Federico II University, 80131 Naples, Italy; 6 UOC of Radiology, Istituto Nazionale Tumori, IRCCS-Fondazione “G. Pascale”, 80131 Naples, Italy; 7 UOC of Oncology, A.O. dei Colli, Monaldi Unit, 80131 Naples, Italy; 8 Molecular Biology and Viral Oncology Unit, Istituto Nazionale Tumori IRCCS “Fondazione G. Pascale”, 80131 Naples, Italy

**Keywords:** neuroendocrine neoplasms, chemotherapy, temozolomide, metronomic treatment, second-line

## Abstract

Background. Platinum-based chemotherapy is the mainstay of front-line treatment of patients affected by pluri-metastatic intermediate/high grade NeuroEndocrine Neoplasms (NENs). However, there are no standard second-line treatments at disease progression. Previous clinical experiences have evidenced that temozolomide (TMZ), an oral analog of dacarbazine, is active against NENs at standard doses of 150 to 200 mg/mq per day on days 1 to 5 of a 28-day cycle, even if a significant treatment-related toxicity is reported. Methods. Metastatic NENs patients were treated at the ENETS (European NeuroEndocrine Tumor Society) center of excellence of Naples (Italy), from 2014 to 2017 with a second-line alternative metronomic schedule of TMZ, 75 mg/m^2^ *per os* “one week on/one week off”. Toxicity was graded with NCI-CTC criteria v4.0; objective responses with RECIST v1.1 and performance status (PS) according to ECOG. Results. Twenty-six consecutive patients were treated. Median age was 65.5 years. The predominant primary organs were pancreas and lung. Grading was G2 in 11 patients, G3 in 15. More than half of patients had a PS 2 (15 vs. 11 with PS 1). The median time-on-temozolomide therapy was 12.2 months (95% CI: 11.4–19.6). No G3/G4 toxicities were registered. Complete response was obtained in 1 patient, partial response in 4, stable disease in 19 (disease control rate: 92.3%), and progressive disease in 2. The median overall survival from TMZ start was 28.3 months. PS improved in 73% of patients. Conclusions. Metronomic TMZ is a suitable treatment for G2 and G3 NENs particularly in PS 2 patients. Prospective and larger trials are needed to confirm these results.

## 1. Introduction

NeuroEndocrine Neoplasms (NENs) are a group of tumors arising from the neuroendocrine cell compartment present in different tissues [1,2]. Their management is complex and depends on tumor grading, differentiation, proliferation index and presence of specific syndromes and/or metastases [3]. The front-line treatment of pluri-metastatic intermediate/high grade NENs is based on systemic platinum-based chemotherapies [4,5]. However, when the disease progresses, there is a lack of evidence for standard second-line treatments.

Temozolomide (TMZ) is an orally active alkylating agent analogue of the dacarbazine. In monotherapy and at the standard doses of 150–200 mg/m^2^ once daily for 5 every 28 days, TMZ showed to be active in pre-treated patients affected by NENs with response rates (RR) of 14% in patient with G1/G2 NENs [6] and a disease control rate (DCR) of 38% in G3 NeuroEndocrine Carcinomas (NECs) [7]. In association with other drugs, namely capecitabine, everolimus, bevacizumab and octreotide, and thalidomide the RR ranges between 17–70% [8,9,10,11,12,13,14,15,16]. The large part of these studies is small (<25 patients) and/or retrospective because of the low incidence of the disease. The most frequent reported all-grade toxicities of TMZ single-agent or combined with other drugs are anemia, leucopenia, thrombocytopenia, hand-foot syndrome and gastrointestinal. However, a discontinuation rate of TMZ up to 55% is reported [16], and in association with everolimus, the treatment with TMZ has been precautionary administrated for a maximum of 6 months in order to reduce toxicity [13].

The use a metronomic schedule of TMZ represents a possible way to reduce toxicity. Metronomic TMZ (mTMZ) consists on lower daily doses with greater frequency of administration. The main biological effects reside on anti-angiogenic activity [17,18,19] and immune-modulation leading to improvement of dendritic cells function [20] and selective depletion of CD4^+^CD25^+^Foxp3^+^ regulatory T cells (Tregs), which are potent immunosuppressive cells within the tumor microenvironment [21,22,23,24].

There are no studies in literature evaluating the activity and safety of mTMZ in advanced pre-treated intermediate/high grade NENs. In this study, we evaluated the efficacy of mTMZ in a consecutive series of 26 NENs patients treated at the ENETS (European NeuroEndocrine Tumor Society) center of Naples.

## 2. Experimental Section

### 2.1. Patients, Treatment and Disease Characteristics

This was a retrospective study approved by the Scientific Directorate (among criteria: Reliable and verifiable source of data, consecutiveness of the cases to reduce biases, adequate follow-up, monocentric radiologic evaluations) of the National Cancer Institute of Naples and conducted at the ENETS Center of Excellence in Naples (Italy). The ENETS center of Naples internal database collects data about NENs’ patients from three different institutions; it was utilized to identify consecutive cases of patients with advanced G2-G3 NENs (Naples, Italy), progressed after a first-line systemic therapy and treated with second-line mTMZ therapy between 2014 and 2017. All patients had progressive and measurable metastatic disease with an Eastern Cooperative Oncology Group Performance Status (ECOG PS) from 0 to 2 and life expectancy greater than three months. Adequate hematological, renal, and hepatic function with laboratory values demonstrating WBC ≥3000/mm^3^, platelet count ≥100,000/mm^3^, hemoglobin >8.0 g/dL, ALT and AST≤ to 3.5 times the upper limit of normal, creatinine ≤1.6 mg/dL, and total bilirubin ≤2.0 mg/dL were also required. Patients were excluded in case of active systemic infections, coagulation disorders or decompensated chronic illnesses. Following the procedures of our Institute, retrospective studies are submitted only to the approval of Scientific Directorate and do not require ethical approval.

The treatment schedule consisted on oral administration of “one week on/one week off” TMZ at 75 mg/m^2^ until unacceptable toxicity or progression. The drug was taken on an empty stomach (1 h before or 2 h after eating), with a full glass of water. Written informed consent was obtained before prescribing and starting therapy. Data about patients and disease characteristics (age, gender, PS, comorbidities, stage), histology (primary tumor site and size, Ki67 status), previous treatments (surgery and/or systemic treatments) were shown in Table 1. The median age was 65.5 years (range: 32–88 years) and the genders were equally represented (13 patients were male and 13 patients were female). Fifteen patients (58%) had an ECOG PS of 2 before starting the second line treatment, while 11 patients (42%) presented with a PS equal to 1. No patient had a PS of 0. Grading is a fundamental characteristic to drive therapeutic choices, G2 NENs were 42% and G3 58%. Among the G3 NENs, 10 out of 15 (67%) had a Ki67 between 20% and 55%. The predominant primary sites were pancreas and lung, whereas the predominant site of metastasis was the liver followed by loco-regional nodes and bone. In half of the patients, metastases were present in a single site, and the liver was the only involved site in the 81% of patients. In contrast, 8 patients (31%) had two different sites of metastasis, and in 5 patients (19%) the sites of metastasis were equal or more than three. The majority of patients (54%) was previously treated with chemotherapy, whereas 31% received Somato Statin Analogues (SSAs) as first line treatment, and 15% received other treatments including immunotherapy or targeted/biologic therapies. Of the 14 patients who received first line chemotherapy, 12 received platinum-based treatments and two non-platinum chemotherapy regimens. In addition, among the chemo-treated patients, 10 (71%) had a G3 NEN but 4 (29%) had a G2 NEN. In the latter patients, the choice to administer chemotherapy was based on the primary site of the NENs and/or on the Ki67: two atypical carcinoids with a Ki67 ≥ 15%, one intracranial neuroendocrine tumor and one NEN of unknown primary origin with a Ki67 of 18%. Of the 8 patients who received SSAs, half received octreotide and half lanreotide.

### 2.2. Activity, Toxicity and Clinical Benefit Evaluations

Tumor assessment was performed every three months through Computed Tomography (CT) scans. Responses to treatment were defined according to RECIST 1.1 (Response Evaluation Criteria in Solid Tumors) [20]. Complete response (CR) was defined as the disappearance of all lesions, and partial response (PR) as a decrease of 30% or more in the sum of the longest diameters. Progressive disease (PD) was defined as either the appearance of new lesions or an increase of 20% or more compared with the minimum sum of longest diameters recorded since the start of treatment. Stable disease (SD) was defined when the sum increased by <20% or decreased by <30% and no new lesions appeared. The objective response rate (ORR) was the sum of CRs + PRs. The disease control rate (DCR) was the sum of CRs + PRs +  SDs.

Adverse events were graded according to the National Cancer Institute Common Terminology Criteria for Adverse Events (NCI-CTCAE version 4) [25]. Physical examination, complete blood counts and blood chemical tests were carried out once a week until the end of the second month and once every 2 weeks thereafter. For each adverse event, the maximum grade per patient was reported. If a patient experienced a toxic effect of any grade on multiple occasions, the event was counted only once. Patients’ toxicities attributable to prior first-line treatment must have recovered to a grade 1 or less (except for alopecia) before starting mTMZ. No grade 3 or 4 toxicities were observed. Grade 2 non-hematologic toxicities were managed by cessation of the drug until resolution to grade 1 and then resuming treatment without a dose reduction. Neither treatment delays nor reductions were applied in case of hematologic grade 1 or 2 or non-hematologic grade 1 toxicities.

The clinical benefit was defined as an improvement of the ECOG PS assessed before starting mTMZ, at 3 and 6 months.

### 2.3. Time-to-Outcome Analysis and Statistical Methods

Data are predominantly descriptive. Progression free survival (PFS) was calculated as the time elapsed from the date of mTMZ initiation to the date of disease progression or death for any cause (whichever occurred first). Patients who were alive with no disease progression were censored at the date of last visit. Overall Survival (OS) was defined as the time from the start of mTMZ administration to the date of death for any cause. Patients who were alive were censored at the date of data analysis. The median PFS (mPFS) and the median OS (mOS) curves were depicted using the Kaplan-Meier method. Exploratory subgroup analyses were done by Log-rank test.

## 3. Results

### Efficacy and Safety

**Figure 1:**
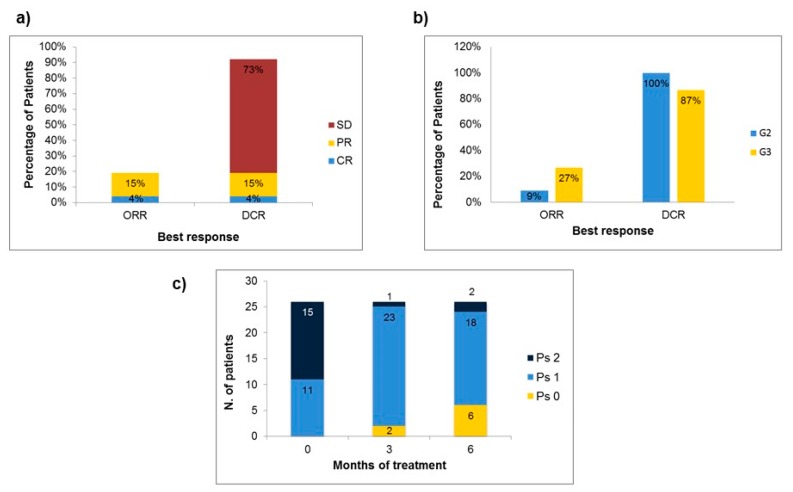
Histogram representations of activity and clinical benefit of mTMZ. Response rates with mTMZ in all patients (**a**) and according to grading (G2 vs. G3) of the tumor (**b**). Improvement of ECOG PS over 3 and 6 months of treatment (**c**). CR = Complete Response; DCR = Disease Control Rate; ORR = Overall Response Rate; PR = Partial Response; PS = Performance Status; SD = Stable Disease.

From 2014 to 2017, twenty-six consecutive patients with advanced G2-G3 NENs in progression after a first line chemotherapy were treated with second-line mTMZ. Characteristics of patients and their disease have been described in the Experimental Section. At last follow-up (median follow-up from mTMZ start: 29 months), 16 (62%) patients were alive, and 8 (31%) were still on treatment with mTMZ. All patients were evaluable for response. The objective response rate (ORR) to second-line mTMZ was 19%, with one complete response (CR) and four partial responses (PR). An additional 73% of patients achieved stable disease (SD) as best response (Table 2 and Figure 1a).

**Figure 2:**
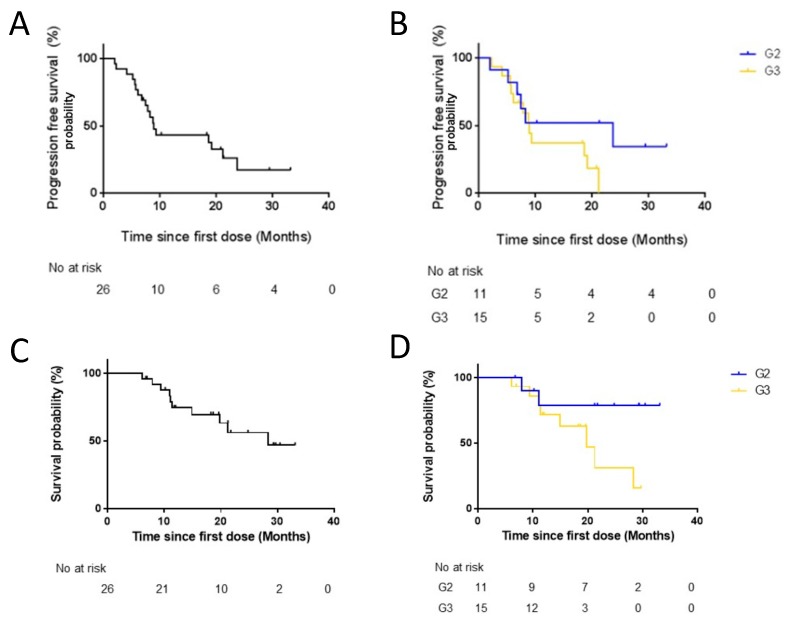
Time-to-outcome analyses. Curves of progression-free survival and overall survival in all patients (**A**,**C**) and according to grading (**B**,**D**).

The overall DCR was 92%. The ORR and DCR in patients with G2 NENs were 9% and 100%, respectively, while for those with G3 NENs the ORR was 27% and the DCR was 87% (Figure 1b). A clinical improvement of the basal PS was reported in 73% of patients (Figure 1c). The mPFS was 9 months and longer for patients with G2 NENs (mPFS: 23.6 months) compared to patients with G3 NENs (mPFS: 8.9 months), although not significant (*p* = 0.16) (Figure 2).

The mOS was 28.3 months in the entire population. The mOS was 19.8 months in patients with G3 NENs and not reached in patient with G2 NENs (*p* = 0.60) (Figure 2). No G3/G4 toxicities were registered (Table 3); no dose reductions were reported. The two most common adverse events were anemia and asthenia (Table 3). The median time-on-TMZ therapy was 12.2 months (95% CI: 11.4–19.6). No patient discontinued treatment for the occurrence of severe adverse events.

## 4. Discussion

Currently, the optimal schedule for TMZ has still not been established. Different schedules have been used in recent trials both in monotherapy and in association with other drugs [6,7,8,9,10,11,12,13,14,15,16]; these studies were heterogeneous in terms of sample size, histology, grading, and number and type of previous treatments (Table S1 in Supplementary Material). Although a significant activity was constantly reported with these schedules, the median time on TMZ was negatively influenced by G3/G4 toxicities [6,13,16] with a discontinuation rate up to 55%. This highlights the need to minimize toxicity. To this regard, albeit retrospective and exploratory, we report the first “hypothesis generating” study with mTMZ 75mg/m^2^ “one week on-one week off” scheme in NENs. The treatment was associated with an ORR of 19% and an overall DCR of 92%; most importantly, no G3/G4 adverse events and no interruptions of treatment for toxicity were registered. In addition, mTMZ determined a clinical benefit through improvement of PS.

Notably, there were no significant relationships between response to therapy and characteristics of patients and disease, including age (≤65 vs. >65 years), sex (male vs. female), PS ECOG (1 vs. 2), site of primary tumor [gastro-intestinal (GI) vs. no-GI], KI-67 level (<20 vs. ≥20%), grading (G2 vs. G3), number of metastatic sites (1 vs. 2 vs. ≥3) and previous treatments (see Table S2 in Supplementary Material). However, we cannot rule out the hypothesis that such evaluations could be affected by the small sample size of our series. In fact, although not significant, responses were more frequently observed in G3 NENs (4 pts) compared to G2 (1 patient). It is well known that grade is associated with cell proliferation rate which is a consistent indicator of chemosensitivity. Unfortunately, high grade tumors are in turn characterized by high genomic heterogeneity with frequent p53, Hedgehog and Notch mutations [26] (associated to drug resistance and plasticity of stem-*like* states), and after an initial response to therapy, they acquire a drug resistant phenotype in a short time. For this reason, very frequently, a discrepancy is observed between higher response rates and shorter progression-free survival in G3 NENs, as occurs in our series.

The clinical advantages of a low-dose administration of TMZ have been explored over the last 20 years and are mainly based on (i) a lower toxicity profile eventually associated (ii) to a better quality of life [27,28,29,30]. An important characteristic of our series was the inclusion of 15 pts with PS ECOG 2 (57.7%) while in previous trials it ranged from 0 to 28% (Table S1 in Supplementary Material). Interestingly, mTMZ was well-tolerated, without any G3/G4 adverse effects, and in 14 out of 15 pts there was an improvement of PS 2 to PS 1 after 3 months of therapy. This suggests that mTMZ might be given to patients with deteriorated PS when the benefit-risk balance is not favorable for more aggressive treatments.

Furthermore, beyond these clinical advantages, mTMZ, but not the conventional scheme, is able to trigger anti-angiogenetic and immune-mediated pathways [17,18,19,20,21,22,23]. NENs are hypervascularised tumors and overexpress a plethora of proangiogenic molecules and related receptors [31,32,33,34,35]. Therefore, given their high dependence from angiogenic pathways, the metronomic schedule, through its predominant anti-angiogenic action, could represent a stronger candidate for NENs treatment. Additionally, metronomic therapy exerts its anti-angiogenetic activity through the increase of the inhibitor thrombospodin-1 (THBS-1) and the inhibition of the hypoxia-inducible factor 1 (HIF-1) [36]. These biologic properties account for a *more* potent and clinically relevant anti-angiogenic than cytotoxic effect of mTMZ. Furthermore, these latter effects could be particularly interesting for combination with mTOR inhibitors (i.e., everolimus). Inhibition of mTORC1 (mTOR Complex 1) causes the loss of a negative feedback loop that activates HIF-1 [37]; therefore, the association of an mTOR inhibitor with mTMZ might preserve the anti-angiogenic activity of this loop.

Notably, the evaluation of O6-methylguanine DNA methyltransferase (MGMT), which repairs the methylation at the O6-position of guanine induced by alkylating agents [38,39,40,41] did not show to be significant in our series. Our group is going to accumulate more data about this issue. On the basis of these clinical results and to further investigate the role of mTMZ in second-line treatment of G3 NENs, a study is currently ongoing at the ENETS center of Naples. This larger and prospective clinical trial named TENEC trial (TEmozolomide in NeuroEndocrine Carcinoma), is supported by ITANET (ITalian Association for NEuroendocrine Tumors) and aims to confirm the efficacy and toxicity results of mTMZ as well as its modulating effects on host’ immune system.

Despite the exploratory and retrospective nature of our study, the efficacy of mTMZ in monotherapy here reported is similar to that shown in other retrospective trials with conventional schedules of TMZ monotherapy; conversely, the toxicity profile is clearly better.

## 5. Conclusions

Our study is a proof of concept that an intermittent schedule of mTMZ at 75 mg/m^2^ can be an effective treatment in advanced G2-3 NENs, a suitable therapeutic option for PS 2 patients as well as a strong candidate also for combination treatments.

## Figures and Tables

**Table 1:** Characteristics of patients and disease.

Characteristics	No.
**Age, years**	
Median	65
Range	32–88
**Gender**	
Male	13
Female	13
**Grading**	
G1	0
G2	11
G3 *	15
**KI-67 level**	
3–20	11
20–55	10
>55	5
**Performance Status**	
0	0
1	11
2	15
**Site of primary tumor**	
Pancreas	5
Lung	5
Stomach	3
Miscellanea	
Head and Neck	2
Small bowel	3
Rectum	1
Gallbladder	1
Cutaneous	1
Unknown Primary Origin	5
**No. of involved metastatic sites**	
1	13
2	8
≥3	5
**Previous treatments**	
Platinum-based treatments	12
Chemotherapy non-platinum based	2
Somatostatin analogues	8
Clinical trials drugs	4

* 3 Large Cell NECs were included, small-cell types were not included.

**Table 2:** Efficacy estimates of second-line mTMZ.

Response to Therapy	No. (%)
Complete Response	1 (3.8)
Partial Response	4 (15.4)
Stable Disease	19 (73.1)
Progressive Disease	2 (7.6)
Median PFS (18 events)	9.0 months
Median OS (10 events)	28.3 months

**Table 3:** Summary of adverse events.

Toxicity	G1		G2		G3/G4	
	No	%	No	%	No	%
Anaemia	11	42.3	13	50.0	0	0.0
Asthenia	9	34.6	12	46.1	0	0.0
Neuropathy	8	30.7	10	38.4	0	0.0
Neutropenia	8	30.7	8	30.7	0	0.0
Nausea	7	26.9	8	30.7	0	0.0
Hyperbilirubinemia	7	26.9	6	23.1	0	0.0
Alkaline phosphatase	3	11.5	0	0.0	0	0.0
Hyperglycaemia	4	15.3	6	23.1	0	0.0
Thrombocytopenia	0	0.0	6	23.1	0	0.0

## Supplementary Materials

The following are available online at https://www.mdpi.com/2077-0383/8/8/1224/s1, Table S1: Characteristics of studies reporting outcomes of TMZ in NENs, Table S2: Clinico–pathological characteristics of patients according to treatment response.
