# Supplementary material for: Safety and Activity of Metronomic Temozolomide in Second-Line Treatment of Advanced Neuroendocrine Neoplasms
Source: J Clin Med. 2019 Aug 15;8(8):1224. doi: 10.3390/jcm8081224 (PMC6723560; doi:10.3390/jcm8081224)
Supplement: Supplementary file 1 [file jcm-08-01224-s001.pdf]

Supplementary Materials

Table S1. Characteristics of studies reporting outcomes of TMZ in NENs.

CI: Confidence Intervals; CR: Complete Response; MGMT: O6-methylguanine DNA methyltransferase; NENs: NeuroEndocrine Neoplasms; NR: Nor Reported; OS: Overall Survival; PR: Partial Response; PS: Performance Status; pts: patients; PD: Progressive Disease; RFA: Radio-Frequency Ablation; RT: RadioTherapy; SD: Stable Disease; TACE: Trans-Arterial Chemo-Embolization; TAE: Trans-Arterial Embolization; TMZ: temozolomide; TTP: time-to-progression.

Year, First Author No. of pts	*Type of NENs*	*PS of pts*	*Line of therapy*	*Association with other drugs*	*Prospective*	*TMZ doses*	*Treatment exposure*	*Toxicity (G3/G4 total events)*	*Objective responses*	*Time–to–outcome results*	*MGMT evaluation*
2006, Kulke MH 29	*Locally unresectable or metastatic neuroendocrine tumors, excluding small–cell carcinoma.*	*ECOG 0: 20 1: 9*	*Pts received from 0 to 4 previous systemic therapies. Previous TACE in 3 pts. Eleven patients received prior therapy with octreotide and remained on octreotide during study.*	*Yes (Thalidomide)*	*Yes (phase II, primary objective was to determine the response rate)*	*150 mg/mq, days 1–7 and days 15–21 every 28 days.*	*Median: 7.3 months (range: 1–23).*	*Hematologic: 29 Non–hematologic: 30*	*28 evaluable pts CR: 1 PR: 6 SD: 19 PD: 2*	*Median TTP: not reached. Median OS: not reached. The 1–year survival rate was 79%, and the 2–year survival rate was 61%.*	*Not done.*
2007, Eklebad S 36	*All advanced or progressing NENs.*	*NR*	*Pts received from 0 to 6 previous systemic therapies.*	*No*	*No*	*100–150 mg/mq, days 1–5 every 28. (dose was escalated to 200 mg/mq/d in 20 pts).*	*Median: 4.5 cycles (range: 0–17).*	*Hematologic: 8 Non–hematologic: 3*	*CR: 0 PR: 5 SD: 19 PD: 12*	*Median TTP: 7 months (95%CI: 3–10). Median OS: 16 months (95%CI: 11–22).*	*Yes (23/36). No predictive power.*
2011, Welin S 25	*Poorly differentiated endocrine carcinoma progressed on first–line chemotherapy.*	*Not detailed. All patients had a PS ECOG of 0–2.*	*Twenty–four patients received cisplatin and etoposide as first line treatment, and 1 patient received docetaxel and doxorubici*	*Yes (TMZ alon –5 pts–or in combination with capecitabine -19 pts–. A subset -7pts– received also*	*No*	*Alone: 150–200 mg/mq, days 1–5 every 28 days. In combination: 150 mg/m2, days 10–14 every 28 days.*	*NR*	*Hematologic: 2 Non–hematologic: 2*	*CR: 1 PR: 7 SD: 10 PD: 7*	*Median TTP: 6 months (95% CI, 4–14). Median OS: 22 months (95% CI, 8–27).*	*Only 1 patient had a MGMT methylation (This patient had a PR for 15 months and an OS of 22 months).*

			n. Seven patients received docetaxel and doxorubicin as 2–line chemotherapy and were given TMZ therapy as 3–line therapy.	bevacizumab. One patient died after 2 months due to a myocardial infarction not suspected to be related to the disease or medication.							
2011, Strosberg JR<br><br>30	Metastatic, well, or moderately differentiated pancreatic endocrine carcinomas.	NR	First–line for advanced disease (pts treated prior octreotide, interferon–α, or hepatic artery embolization were included).	Yes (Capecitabine)	No	200 mg/mq, days 10–14 every 28 days.	Median: 8 cycles (range 3–23).	Hematologic: 2<br>Non–hematologic:<br>2	CR: 0<br>PR: 21<br>SD: 8<br>PD: 1	Median TTP: 18 months (95% CI: 9–31)<br><br>2–year OS: 92% (95% CI, 72–98).	Not done.
2012, Koumarianou A<br><br>15	advanced NETs (Ki67 < 20%) who progressed after at least one regimen of chemotherapy	NR	Pts received from 1 to 3 previous systemic therapies. Other previous therapies were: interferon (7), octreotide long–acting release (15) Everolimus (1), radiolabelled peptides (2), surgery (6).	Yes (Bevacizumab and octreotide long–acting release.	Yes (pilot study to assess toxicity and activity)	Continuous standard daily dose of 100 mg before bedtime.	Median: 12 cycles (range 4–20).	Hematologic: 0<br>Non–hematologic: 1	14 evaluable pts<br><br>CR: 1<br>PR: 8<br>SD: 3<br>PD: 2	Median TTP: 36 weeks (range: 10–60 weeks, 95% CI: 25.2–41.8).	Not done.
2012, Chan JA<br><br>34	Metastatic or locally unresectable NETs (carcinoids	ECOG<br>0: 12<br>1: 20<br>2: 2	Pts received from 0 to "2 or more" previous	Yes (Bevacizumab)	Yes (phase II study, primary objectives were	150mg/mq, days 1–7 and days 15–21 every 28 days.	Median: 4 cycles (range 1–39).	Hematologic: 29<br>Non–hematologic: 13	31 evaluable pts<br><br>CR: 0<br>PR: 5	Median TTP: 11.0months	Not done

	and pNETs), excluding small–cell carcinoma.		systemic therapies including octreotide, chemotherapy, interferons, sunitinib. Previous TAE in 11 pts, RFA in 3, RT in 4.		activity and toxicity)				SD: 22<br>PD: 4	(95%CI, 7.3–Not reached).<br><br>Median OS: 33.3 months (95%CI, 13.4–41.7).	
2012, Holsen IH<br><br>28	Metastatic NEC (Ki–67 > 20%).	ECOG<br>0–1: 22<br>2: 6	Second–line after previous exposure to carboplatin and etoposide.	No	No	200 mg/mq, days 1–5 every 28 days.	Median: 3 cycles (range 1–12).	Hematologic: 3<br>Non–hematologic: NR	16 evaluable pts<br><br>CR: 0<br>PR: 0<br>SD: 10<br>PD: 6	Median TTP: 2.4 months.<br><br>Median OS: 3.5 months.	Not done.
2013, Saif MW<br><br>7	Metastatic pNETs	ECOG<br>0: 1<br>1: 4<br>2: 2	Pts received from 1 to 3 previous systemic therapies. Previous TAE/TACE in 4 pts. One resection of PT.	Yes (capecitabine)	No	200 mg/mq, days 10–14 of a 28–day cycle	NR	Hematologic: 1<br>Non–hematologic: 1	CR: 0<br>PR: 3<br>SD: 2<br>PD: 2	Median TTP: 12 months (range: 10–16).<br><br>Median OS: 24 months. (range: NR)	Not done.
2013, Chan JA<br><br>43	Low– or intermediate– grade metastatic or locally unresectable pNETs.	ECOG<br>0: 20<br>1: 23	Number of previous sistemic terapies (other than octreotide) were 0 in 77%, 1 in 16% and 2 in 7% of pts. One patients received also TACE, 3 pts RT.	Yes (everolimus)	Yes (phase I/II, primary objective was to determine the response rate).	150 mg/mq, days 1–7 and days 15–21 every 28 days.	Median: 8.5 cycles (range 1–28).	Hematologic: 36<br>Non–hematologic: 29	40 evaluable pts<br><br>CR:0<br>PR: 16<br>SD: 21<br>PD: 3	Median TTP: 15.4 months (95% CI, 9.4–20.4).<br><br>Median OS was not reached.	Not done.
2013, Fine RL<br><br>18	Metastatic, well differentiated neuroendocrine cancers.	ECOG<br>0: 4<br>1: 9<br>2: 5	All pts progressed on Sandostatin LAR 60 mg/month. Pts received a median of 2 previuos chemotherapy lines (range: 1–5). Previous	Yes (capecitabine)	No	150–200 mg/mq, days 10–14 every 28 days.	NR	Hematologic: 2<br>Non–hematologic: 1	18 evaluable pts<br><br>CR: 1<br>PR: 10<br>SD: 4<br>PD: 3	Median TTP: 14.0 months (range: 4.2–18).<br><br>Median OS: 83 months (range 18.5–140).	Not done.

			TACE: 9. Previous surgery: 3.								
2013, Saranga–Perry V *3*	*Metastatic neuroendocrine tumors of the thymus.*	*NR*	*Patient 1: radiation concurrent with cisplatin and etoposide, cytoreductive syrgery, bilateral adrenalectomy for palliation of hypercortisolism, octreotide LAR, further chemotherapy with carboplatin and paclitaxel. Patient 2: octreotide LAR and interferon–alpha, Patient 3: pasireotide LAR.*	*Yes (capecitabine)*	*No*	*170–190 mg/mq days 10–14 every 28 days.*	*Patient 1: 19 cycles. Patient 2: 12 cycles–on treatment without dose modifications. Patient 3: 3 cyces–on treatment without dose modifications.*	*Hematologic: 0 Non–hematologic: 1*	*Patient 1: PR Patient 2: PR Patient 3: SD*	*Patient 1 TTP: 28 months, alive. Patient 2, alive without progression. Patient 3, alive without progression.*	*Not done.*

Table S2. Clinico–pathological characteristics of patients according to treatment response.

CR: complete response; CT:chemotherapy; GI: Gastro–Intestinal; PR: partial response.

		Responses (CR+PR)		*P**
**Characteristics**	**No. (%)**	**Yes**	**No**	
**Age, years**				
≤ 65	13 (50.0)	3	10	
> 65	13 (50.0)	2	11	0.6256
**Gender**				
Male	13 (50.0)	3	10	
Female	13 (50.0)	2	11	0.6256
**Grading**				
G1	0 (0.0)	0	0	
G2	11 (42.3)	1	10	
G3	15 (57.7)	4	11	0.2706
**KI–67 level**				
3–20	11 (42.3)	1	10	
20–55	10 (38.5)	2	8	
> 55	5 (19.2)	2	3	0.3464
**Performance Status**				
0	0 (0.0)	0	0	
1	11 (42.3)	2	9	

2	15 (57.7)	3	12	0.9093
**Site of primary tumor**				
GI	13 (50.0)	2	11	
Non–GI	13 (50.0)	3	10	0.6256
**No. of involved metastatic sites**				
1	13 (50.0)	3	10	
2	8 (30.8)	2	6	
≥ 3	5 (19.2)	0	5	0.4758
**Previous treatments**				
Platinum–based CT	12 (46.1)	3	9	
Non–platinum based CT	2 (7.7)	0	2	
Somatostatin analogues	8 (30.8)	1	7	
Clinical trials drugs	4 (15.4)	1	3	0.7886

* At Chi–square test with Yates correction for small datasets.
